# Assessing microglial phagocytosis of apoptotic neurons *in vitro*: a multimodal approach

**DOI:** 10.3389/fnins.2026.1813556

**Published:** 2026-08-12

**Authors:** Anthony Mukwaya, Zhuoran Yin, Ryan Yarcusko, George Baldwin, Nasir Uddin, Harshini Venkat, Yixi Xue, Natalia S. Van Arnam, Hio Tong Kam, Dong Feng Chen, Susanne Krasemann, Anton Lennikov, Milica A. Margeta

**Affiliations:** 1 Department of Ophthalmology, Schepens Eye Research Institute of Mass Eye and Ear, Harvard Medical School, Boston, MA, United States; 2 Department of Pathology, University of California, San Francisco, CA, United States; 3 Clem Jones Centre for Regenerative Medicine, Faculty of Health Sciences & Medicine, Bond University, Gold Coast, QLD, Australia; 4 Institute of Neuropathology, University Medical Center Hamburg-Eppendorf, Hamburg, Germany; 5 Department of Ophthalmology and Visual Neuroscience, University of Minnesota, Minnespolis, MN, United States

**Keywords:** apoptosis, *in vitro*, microglia, phagocytosis, phosphatidylserine

## Abstract

Microglia are the resident immune cells of the central nervous system, responsible for defending against infections, responding to tissue damage, and maintaining homeostasis. Dysregulation of their phagocytic activity has been implicated in many neurodegenerative diseases of the brain and the eye, including Alzheimer's disease, multiple sclerosis, glaucoma, and age-related macular degeneration. Past work has shown that brain and retinal microglia switch to a disease-associated molecular phenotype (DAM) following phagocytosis of apoptotic neurons *in vivo*, but it is technically challenging to perform these assessments and isolate phagocytic cells for downstream analysis. Although several protocols exist for isolating and culturing microglia to evaluate their phagocytic activity *in vitro*, these approaches often fail to mimic the pathophysiology of neurodegeneration, where dysregulated phagocytosis of damaged or apoptotic neurons is a key feature. Here we describe a protocol for evaluating phosphatidylserine-mediated phagocytosis by primary mouse brain microglia *in vitro*. The procedure involves isolating primary mouse brain microglia, inducing apoptosis in a feeder neuronal cell line, labeling apoptotic cells with a fluorescent dye, feeding the labeled cells to the microglia, and analyzing phagocytosis using several complementary approaches. This protocol enables an accessible and high-throughput assessment of microglial phagocytic activity using a biologically relevant stimulus, offering improved insights into the regulation of this critical immune process in a disease-relevant context.

## 1 Introduction

Microglia, the resident immune cells of the central nervous system (CNS), play essential roles in maintaining neuronal health and function. They participate in antigen presentation, secretion of signaling molecules, and phagocytosis of cellular debris. Microglial phagocytosis is vital for both normal development and tissue homeostasis. During CNS development, microglia mediate synaptic pruning and engulf apoptotic neural progenitor cells, processes critical for refining neuronal circuitry and establishing proper brain connectivity (Paolicelli et al., 2022). Additionally, microglia in the embryonic retina are highly phagocytic, engulfing non-apoptotic retinal ganglion cells (RGCs) to regulate neuronal density (Anderson et al., 2019). In the adult retina, microglia maintain homeostasis by continuously surveying the microenvironment, clearing photoreceptor debris and apoptotic cells, and modulating synaptic activity to preserve visual function (Rathnasamy et al., 2019).

The tightly regulated phagocytic pathway consists of three key steps: recognition, engulfment, and degradation (Sierra et al., 2010). First, apoptotic cells secrete pro-inflammatory mediators, cytokines such as fractalkine, and ATP (Medina and Ravichandran, 2016). These apoptotic cell ligands are recognized by several constitutively expressed microglial cell surface receptors, including complement receptors (CR3), Triggering Receptor Expressed on Myeloid cells 2 (TREM2), TAM receptors (Tyro3, Axl, and Mer) and Pyrimidinergic receptor P2Y6 (Hadas et al., 2012; Jones, 2013; Lew et al., 2014). Activation of these microglial surface receptors initiates the formation of phagocytic cups on the cell membrane. Binding of the activated microglial phagocytic receptors to apoptotic cells is mediated by cell surface pro-engulfment molecules such as phosphatidylserine (PS) and heat-shock proteins (Fu et al., 2014). The bound apoptotic cell is ultimately internalized and degraded enzymatically within the phagolysosome.

While phagocytosis is critical for normal development and homeostasis in the CNS, dysregulation of phagocytosis is associated with many neurological diseases affecting the brain and the eye. In Alzheimer's Disease (AD), downregulation of the phagocytosis of harmful aggregates such as amyloid-β plaques and neurofibrillary tau tangles contributes to disease progression (Sarlus and Heneka, 2017). In addition, upregulation of microglial phagocytosis of living neurons and synapses can also be harmful (Vilalta and Brown, 2018). Prior work suggests that dysregulated phagocytosis is mediated by the excessive release of pro-recognition and pro-engulfment signals, or the loss of anti-engulfment signals by healthy neurons (Butler et al., 2021). For example, knockout of pro-engulfment genes *C1q* and *C3* reduced excessive synaptic pruning in a mouse model of AD (Hong et al., 2016). Further, in a mouse model of retinitis pigmentosa, activated retinal microglia were found to contribute to retinal degeneration by phagocytosing degenerating rods upregulating pro-engulfment genes (Zhao et al., 2015).

Recent advances in bulk and single cell RNA sequencing have shown that activated microglia acquire a disease associated molecular phenotype (DAM) in mouse models of AD, multiple sclerosis, amyotrophic lateral sclerosis, photoreceptor degeneration, and glaucoma (Keren-Shaul et al., 2017; Krasemann et al., 2017; O'Koren et al., 2019; Margeta et al., 2022). This molecular signature is characterized by downregulation of homeostatic genes and upregulation of Apolipoprotein E (*ApoE*), Galectin-3 (*Lgals3*), Osteopontin (*Spp1*), and glycoprotein non-metastatic melanoma protein B (*Gpnmb*). Notably, microglia can also acquire the DAM signature after phagocytosing injected, fluorescently labeled apoptotic neurons in the brain and the eye *in vivo* (Krasemann et al., 2017; Margeta et al., 2022). However, *in vivo* assays are technically challenging and limit the number of cells that can be isolated for downstream analysis.

*In vitro* phagocytosis assays, on the other hand, have provided invaluable mechanistic insights. Well-established protocols exist for the isolation, culture, and quantification of phagocytosis in primary microglia (Lee and Tansey, 2013), monocytes (Cline and Lehrer, 1968), and macrophages (Davies and Gordon, 2005). However, while in neurodegenerative diseases phagocytes engulf cellular debris, apoptotic cells, and damaged cells, most *in vitro* phagocytosis assays have used other substrates as stimuli, including Zymosan particles (Adams, 2012), glass beads (Joffe et al., 2020), nano particles (Potter et al., 2020), and fluorescent beads (Layman et al., 2022). These stimuli do not accurately mimic the complex *in vivo* microenvironment of the CNS and may fail to evoke phagocytic mechanisms that replicate *in vivo* phagocytic pathways, which makes it difficult to interpret results in the context of neurodegeneration.

Here we describe a protocol for evaluating phosphatidylserine (PS)-mediated *in vitro* phagocytosis by freshly isolated primary mouse brain microglia. The procedure involves isolating primary mouse brain microglia as previously described (Lee and Tansey, 2013), induction of apoptosis in a feeder neuronal cell line using UV irradiation to expose PS residues (Yu et al., 2003; Berglund et al., 2004), labeling the apoptotic cells with a fluorescent dye, feeding the labeled apoptotic cells to the isolated microglia in culture, and analyzing phagocytosis using several complementary approaches including Fluorescent Activated Cell Sorting (FACS), immunocytochemistry (ICC), and *in vitro* timelapse recording. PS exposure and the use of a feeder cell line are employed as a well-controlled system to demonstrate a biologically relevant phagocytic stimulus. The overall workflow is not limited to this specific substrate or engulfment signal and can be readily adapted to study alternative phagocytic targets, signaling mechanisms, or disease-relevant contexts. As such, this protocol provides an accessible, scalable, and flexible framework for assessing microglial phagocytic capacity to address specific scientific questions relevant to neurodevelopment, homeostasis, and neurodegeneration.

## 2 Materials and equipment

### 2.1 Consumables

- 1μl, 20 μl, 200 μl, and 1000 μl pipette tips
- 10 ml and 50 ml serological pipettes
- 1.7” scalpel blades
- Super frost microscope slides
- 24 × 24 mm coverslips
- 50 ml syringes
- 24-gauge needles
- 12-well and 24-well petri dishes
- T75 cell culture flask
- FACS tubes
- 1.5 ml, 15 ml and 50 ml tubes
- Cell counting slides
- Millicell EZ slides (Sigma, MO, USA, Cat. No.: PEZGS0816)

### 2.2 Reagents

- Phosphate buffered saline (PBS) (ThermoFisher Scientific, MA, USA, Cat. No.: 10010-023)
- 10X PBS Molecular Biology Grade (ThermoFisher Scientific, MA, USA, Cat. No.: 46-013-CM)
- Percoll^TM^ plus (Cytiva, MA, USA, Cat. No.: 17544501)
- DMEM (1X) (ThermoFisher Scientific, MA, USA, Cat. No.: 11885-084)
- Mouse-Recombinant carrier free Macrophage Colony-Stimulating Factor (M-CSF) (R&D Systems, MN, USA, Cat. No.: 416-ML-010/CF)
- TE Trypsin/EDTA solution (1X) (Gibco, MA, USA, Cat. No.: R001100)
- Trypan Blue Stain 0.4% (ThermoFisher Scientific, MA, USA, Cat. No.: T10282)
- Bovine Serum Albumin (Sigma, MO, USA, Cat. No.: A9647-50G)
- Normal Donkey Serum (Jackson Immunoresearch, PA, USA, Cat.No.: 071-00-121)
- Triton X-100 solution 10% (Sigma, MO, USA, Cat. No.: 93443-100ML)
- Tween-20 (Sigma, MO, USA, Cat. No.: P9416-100ML)
- Alexa Fluor^TM^ 405 NHS ester (succinimidyl ester) (ThermoFisher Scientific, MA, USA, Cat. No.: A30000)
- Prolong Diamond Antifade Mountant (ThermoFisher Scientific, MA, USA, Cat. No.: P36970)
- Paraformaldehyde (PFA) 4% (ThermoFisher Scientific, MA, USA, Cat. No.: J61899. AP)
- HBSS (+) Calcium and (+) Magnesium (ThermoFisher Scientific, MA, USA, Cat. No.: 14025092)
- pHrodo™ Red, succinimidyl ester (pHrodo™ Red, SE) (ThermoFisher Scientific, MA, USA, Cat. No.: P36600)
- Guinea pig anti-Iba1 (Synaptic Systems, G?ttingen, Germany, Cat. No.: 234 308)
- Alexa Fluor^TM^ 488 donkey anti-guinea pig (Synaptic Systems, G?ttingen, Germany, Cat. No.: 706-545-148)
- Heat-inactivated FBS (ThermoFisher Scientific, MA, USA, Cat. no.: 10270-106)
- 10,000 U/ml penicillin, 10,000 μg/ml streptomycin (ThermoFisher Scientific, MA, USA, Cat. No.: 15140-122)
- Cell staining buffer (Biolegend, SD, CA, Cat. No.: 420201)
- Annexin V binding buffer (Biolegend, SD, CA, Cat. No.: 422201)
- Purified annexin V (Biolegend, SD, CA, Cat. No.: 640901)
- PE anti-Tmem119 (Biolegend, SD, CA, Cat. No.: 853315)
- Propidium iodide (Sigma, MO, USA, Cat. No.: P4170-10MG)
- Rabbit anti-P2ry12 (Cell Signaling Technology, MA, USA, Cat. No.: 69766)
- Chicken anti-Iba1 (Synaptic Systems, G?ttingen, Germany, Cat. No.: 234009)
- Guinea pig anti-Tmem119 (Synaptic Systems, G?ttingen, Germany, Cat. No.: 400004)
- Guinea pig anti-Apoe (Synaptic Systems, G?ttingen, Germany, Cat. No.: 465004)
- Rat anti-Galectin-3 (Biolegend, SD, CA, Cat. No.: 125401)
- Goat anti-OPN (R&D Systems, MN, USA, Cat. No.: AF808)

#### 2.2.1 Staining solutions for flow cytometry

- FACS buffer: 0.2% BSA in HBSS.
- Blocking buffer: 2% BSA in HBSS.

#### 2.2.2 Solutions for cell staining

- 2% PFA (20 ml)
- Blocking buffer (100 ml): 10% normal donkey serum, 0.25% triton-X, 0.25% tween-20, and 1% BSA in 1X PBS.
- Staining buffer (100 ml): 2% normal donkey serum, 0.25% triton-X, 0.25% tween-20 and 1% BSA in 1X PBS.

#### 2.2.3 Solutions for microglia cell isolation

- 100% isotonic Percoll: 5ml of 10X PBS and 45 ml of Percoll^TM^ Plus.
- 70% Percoll: 35 ml of the isotonic Percoll and 15 ml of HBSS.
- 37% Percoll: 15 ml of the isotonic Percoll and 25 ml of HBSS.

#### 2.2.4 Complete microglia culture media

- DMEM (ThermoFisher Scientific, MA, USA, Cat. No.: 11885-084) supplemented with 10% heat-inactivated FBS and 1% 10,000 U/ml penicillin, 10,000 μg/ml streptomycin.
- Dissolve 5 μg of M-CSF in 100 μl PBS to make a working solution of 50 μg/ml and make aliquots. Add 10 μl of the working solution for every 10 ml of complete culture media.

#### 2.2.5 Neuroblastoma cell line culture media

- DMEM (ThermoFisher Scientific, MA, USA, Cat. No.: 11885-084) supplemented with 10% heat-inactivated FBS and 1% 10,000 U/ml penicillin, 10,000 μg/ml streptomycin.

#### 2.2.6 Solutions for Annexin V staining of apoptotic cells

- Cell Staining Buffer (Biolegend, SD, CA, Cat. No.: 420201)
- Annexin V Binding buffer (Biolegend, SD, CA, Cat. No.: 422201)
- Purified Annexin V (Biolegend, SD, CA, Cat. No.: 640901 Bio)

### 2.3 Equipment

- Cell counter (Thermofisher Scientific, MA, USA, Cat. No.: AMQAX2000)
- Water bath with adjustable temperature (Thermofisher Scientific, MA, USA, Cat. No.: FSGPD02)
- UV transilluminator (Avantor, PA, USA, Cat. No.: 89131-448)
- AXIO Observer Inverted Fluorescent Microscope (ZEISS, Oberkochen, Germany)
- Phase contrast microscope (Thermofisher Scientific, MA, USA, Cat. No.: 01-672-695)
- NovoCyte Flow Cytometer (Agilent Technologies, CA, USA)
- Humidified CO_2_ Incubator (Thermofisher Scientific, MA, USA, Cat. No.: 51026332)
- Pipettes (P10, P20, P200 and P1000)
- 7 ml Dounce tissue grinder set (DWK, NJ, USA, Cat. No.: K885300-0007)
- Orbital shaker (DLAB Scientific, Beijing, China, Cat. No.: 8072210300)
- Centrifuge for 1.5 ml Eppendorf tubes (Thermofisher Scientific, MA, USA, Cat. No.: 75002446)
- Centrifuge for 15 ml and 50 ml tubes (Thermofisher Scientific, MA, USA, Cat. No.: 75009525)
- EVOS M7000 Live Imaging Microscope (Thermofisher Scientific, MA, USA)

### 2.4 Data analysis software

- FlowJo (Treestar, Inc. Ash, OR USA)
- ImageJ (Fiji)

### 2.5 Animals

- CX3CR1-EYFP (B6.129P2(Cg)-*Cx3cr1*^*tm*2.1(*cre*/*ERT*2)*Litt*^ hfill/WganJ, JAX# 021160): Purchased from Jackson Laboratory (Bar Harbor, ME. USA).

- C57BL/6J (JAX# 000664): Purchased from Jackson Laboratory (Bar Harbor, ME. USA).

## 3 Methods

### 3.1 Experimental workflow

**Figure 1:**
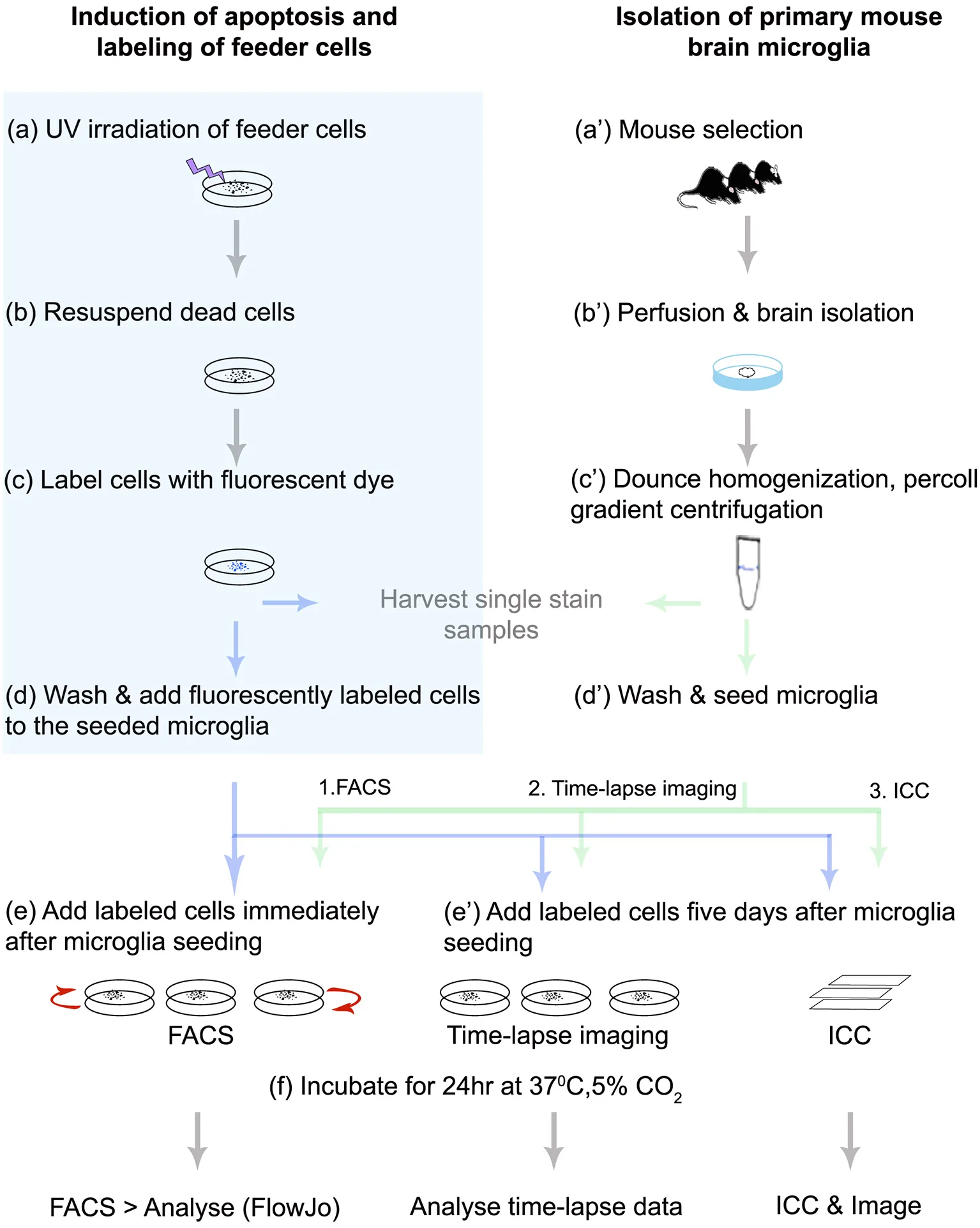
Schematic representation of the workflow for the *in vitro* microglia phagocytosis assay, which involves inducing apoptosis in feeder cells (a-e) and the culture of the primary mouse brain microgila (a′-e′). Apoptotic feeder cells and microgila are co-cultured, and phagocytosis is analyzed by fluorescents activated cell sorting, time-lapse imaging and immunocytochemistry (f).

Primary mouse brain microglia were isolated from C57BL/6J or CX3CR1-EYFP mice and combined with labeled, apoptotic SH-SY5Y neuroblastoma cells. Phagocytosis was quantified using FACS, ICC, and time-lapse imaging (Figure 1).

### 3.2 Isolation of primary mouse brain microglia

The Institutional Animal Care and Use Committee at MEEI Schepens Eye Research Institute approved all animal experimental procedures. Animals were bred at Schepens Eye Research Institute, kept in a 12/12h day light cycle, and provided with food *ad libitum*. 6 to 8-week-old mice were used for brain microglia isolation, as previously shown (Lee and Tansey, 2013; Chakrabarti et al., 2023). Briefly, the animals were euthanized by CO_2_ inhalation and perfused with room temperature HBSS until the liver lost color. The whole brain was quickly harvested, and the cerebellum was carefully dissected and removed. The remaining brain tissue was immersed in ice-cold HBSS in a 15 ml tube.

The tissue was mechanically dissociated with the Dounce homogenization method. First, the tissue was transferred into a 7 ml glass mortar containing 5 ml of HBSS. 10 half strokes were applied using pestle “A”, followed by 10 full strokes with pestle “B”. The homogenate was transferred into a 15 ml tube and centrifuged to pellet (340 x g, 7 mins, acceleration 9, deceleration 9, 4°C). The supernatant was decanted, and the homogenate was thoroughly resuspended in 6 ml of room temperature 70% Percoll solution. A layer of 37% Percoll solution was pipetted on top of the 70% Percoll solution using the “gravity” setting of a serological pipette. Cells were separated across the density gradient by centrifugation (800 x g, 25 mins, acceleration 3, deceleration 2, 23°C).

Up to 3 ml of the interface (cloudy suspension) containing the microglial cells was transferred into a new 15 ml tube using a 1 ml pipette. The collected cells were washed with 5 ml of 0.2% BSA in HBSS and centrifuged to pellet (340 x g, 7 mins, acceleration 9, deceleration 9, 4°C) (Figure 1). The cell pellet was resuspended in complete microglia cell culture media containing M-CSF. The microglia were maintained in cell culture media containing M-CSF during the first 5 days in culture to promote cell growth and survival. During the phagocytosis assay, non-M-CSF containing cell culture media was used to ensure maximal microglial activation and phagocytic response. The number of cells and cell viability was then determined using the trypan blue exclusion assay (Strober, 1997). Microglial yield was approximately 6,000 cells per brain.

### 3.3 Maintenance of the SH-SY5Y cell line

The neuroblastoma feeder cell line SH-SY5Y was acquired from the American Type Culture Collection (ATCC CRL-2266). The cells were grown in complete culture media defined in section 2.2.4 and maintained at 37°C, 5% CO_2_, and 70% humidity. Culture media was changed once per week, and cells were split at 80% confluency.

### 3.4 Procedure

#### 3.4.1 Apoptosis induction

**Day 1**

1. When the SH-SY5Y culture reaches 80% confluency in a T75 flask, wash the cells with 5 ml of room temperature PBS.
2. Decant the PBS, add 1 ml of Trypsin/EDTA (TE), and incubate at 37°C for 3 minutes to detach the cells.
3. Stop trypsinization by adding 3 ml of complete culture media to the flask.
4. Pellet the cells by centrifuging (340 x g, 5 mins, 4°C).
5. Resuspend cell pellet in 5 ml of fresh complete culture media in a T75 flask.
6. UV irradiate the flask of cells for 15 min with the UV transilluminator. This results in an estimated maximum UV dosage of 44 J/cm^2^, although energy loss through absorption may lower actual UV dosage to 25-30 J/cm^2^.
7. Transfer the flask containing the UV irradiated cells into a humidified cell culture incubator and incubate for 24h at 37°C, 5% CO_2_, and 70% humidity.

**Day 2**

1. Resuspend the UV-irradiated cells by flushing using a 10 ml serological pipette.
2. Transfer the cell suspension into a 15 ml tube and centrifuge to pellet (340 x g, 5 mins, 4 °C).
3. Resuspend the UV-irradiated cell pellet in 1 ml of room temperature PBS.
4. Determine the number of cells and cell viability using the trypan blue exclusion assay.
5. Resuspend the UV-irradiated cells in neuroblastoma culture media at a concentration of 1,000 cells/μl.

#### 3.4.2 Apoptotic cell labelling

1. Aliquot the UV-irradiated cells into a 50 ml tube.
2. Pellet the cells by centrifuging (340 x g, 5 mins, 4°C) and resuspend the cell pellet in 1 ml of room temperature PBS.
3. Add 2 μl fluorescent dye (Alexa Fluor 405-NHS) to cells and incubate for 15 mins at 37°C.
4. Pellet the labeled cells by centrifuging (340 x g, 5 mins, 4°C).
5. Wash the labeled cells by resuspending the pellet in 20 ml of room temperature PBS.
6. Pellet the labeled cells by centrifuging (340 x g, 5 mins, 4°C).
7. Resuspend the cell pellet in neuroblastoma culture media at a concentration of 1,000 cells/μl.
8. Aliquot 20,000 UV-irradiated, labeled cells into a pre-blocked FACS tube and store at 4°C. This sample will be used for setting the YFP gate during cell sorting.

#### 3.4.3 Blocking of exposed PS by annexin V

1. Induce apoptosis and label a fresh flask of the feeder cells as described in sections 3.4.1−3.4.2. respectively.
2. Wash the UV-irradiated, labeled cells twice with cold Biolegend Cell Staining Buffer.
3. Pellet the labeled cells by centrifuging (340 x g, 5 mins, 4°C).
4. Resuspend the cell pellet in Annexin V binding buffer at a concentration of 1x10^6^ cells/ml. Annexin V binding buffer contains 2.5 mM Ca^2+^ and is optimized for Annexin V binding.
5. Add 5 μg of purified Annexin V/100,000 cells, which binds to the exposed PS of the apoptotic cells.
6. Gently vortex the cell suspension and incubate for 15 mins at room temperature.
7. Pellet the labeled cells by centrifuging (340 x g, 5 mins, 4°C).
8. Resuspend the labeled cells in neuroblastoma culture media at a concentration of 1,000 cells/μl.

#### 3.4.4 Assessment of phagocytosis by FACS

1. Once the feeder cells are prepared, isolate primary mouse brain microglia as described in section 3.2.
2. Seed the isolated cells into the wells of a 12-well tissue culture flask, in three technical replicates per condition.
3. Add labeled, apoptotic feeder cells into each well containing microglia at a ratio of 2:1 (Cosker et al. 2021).
4. For a negative control, add labeled, apoptotic, Annexin V-treated feeder cells into separate wells containing microglia at a ratio of 2:1.
5. Incubate the cell mixture for 24h at 37°C, 5% CO_2_, and 70% humidity with gentle rocking.

**Day 3**

1. Gently remove media using a P1,000 pipette without disturbing the cells.
2. Detach the cells by flushing twice with 300 μl of 0.2% BSA solution.
3. Transfer the cells into a 15 ml pre-blocked tube.
4. Pellet the cells by centrifuging (340 x g, 5 mins, 4°C).
5. Resuspend the cell pellet in 300 μl PBS with 0.2% BSA.
6. Transfer the cells into a pre-blocked FACS tube.

##### 3.4.4.1 Fluorescence activated cell sorting (FACS) for phagocytic and non-phagocytic microglia

1. Set the individual gates using the single stained samples prepared in 3.4.2.
2. Run the test samples using the defined settings and collect the sorted cells into two separate individual collection tubes, one for phagocytic cells and another for non-phagocytic cells.
3. Centrifuge the sorted cells, remove the supernatant, and store the pellet at −80 °C for downstream analysis.

##### 3.4.4.2 Data acquisition and analysis

1. Once the samples are sorted, transfer the FACS files to FlowJo software.
2. Plot FSC vs. SSC to exclude debris.
3. Plot FSC-H vs. FSC-A gating for singlets.
4. Plot SSC-H vs. SSC-A to identify cells based on their complexity.
5. Plot YFP vs. SSC-A to identify the microglial cell population.
6. Plot 405 vs. SSC-A to identify phagocytic microglia.

#### 3.4.5 Assessment of phagocytosis by ICC

**Day 1**

1. Isolate primary mouse brain microglia as detailed in section 3.2. and prepare UV-irradiated, labeled apoptotic neurons as detailed in sections 3.4.1-2.
2. Seed the isolated microglia into individual wells of a 4-well EZ slide, preferably one mouse brain/well.
3. Add 250 μl of complete microglia culture media to each well and incubate at 37°C, 5% CO_2_, and 70% humidity for 3 days.

**Day 4**

1. Replace 50% of the culture media with fresh complete microglia culture media and incubate at 37°C, 5% CO_2_, and 70% humidity for 24h.

**Day 5**

1. Replace 100% of the culture media with fresh microglia culture media, without M-CSF.
2. Add the prepared UV-irradiated-labeled apoptotic neurons to the microglia at a ratio of 2:1 and incubate the cell mixture 37°C, 5% CO_2_, and 70% humidity for 24h.

**Day 6**

1. Drain the culture media from the culture slide by gently inverting the slide onto tissue paper.
2. Gently add 250 μl of room temperature PBS to each well to wash off the loose dead neurons and immediately invert the slide to drain the PBS.
3. Gently add 250 μl of 2% PFA to each well and incubate at room temperature for 5 minutes. Invert the slide to drain the PFA into a collection container.
4. Gently add 250 μl of room-temperature PBS to each well to wash the slide, then invert the slide to drain the PBS.
5. Gently add 250 μl of blocking buffer to each well and incubate for 1 hour at room temperature. Invert the slide to drain.
6. Gently add primary antibody: Guinea pig (Gp) anti-Iba1 (1:200) diluted in staining buffer, and incubate over night at 4°C.

**Day 7**

1. Invert the slide to drain the unbound primary antibody.
2. Gently add 250 μl of room-temperature PBS to wash the slide, then immediately invert the slide to drain the PBS.
3. Gently add 250 μl secondary antibody (Alexa Fluor^TM^ 488 donkey anti-guinea pig: 1:800 in staining buffer) and incubate at room temperature for 2h.
4. After the incubation period, invert the slide to drain the unbound secondary antibody.
5. Gently add 250 μl room temperature PBS to each well to wash off non-specific secondaries.
6. Invert the slide to drain the PBS and disassemble the EZ slide assemblage.
7. Apply mounting media (without DAPI) onto a coverslip and lay over the stained cells on the slide.
8. Apply nail polish to the edges of the coverslip to ensure it is firmly attached to the slide.
9. Airdry the mounted slide in the dark for 15 mins at room temperature and image using a fluorescent microscope at 20x magnification.

##### 3.4.5.1 Quantifying phagocytosis with ICC

1. Import the raw image files (.lif) into ImageJ (Fiji) (Schindelin et al., 2012).
2. Adjust the color contrast for each channel separately if necessary.
3. Select the Cell Counter plugin, initialize, and ‘select type'.
4. Manually count phagocytic microglia (neurons in contact or inside microglia), and non-phagocytic microglia (microglia not in contact with neurons).
5. Express the number of phagocytic cells as a percentage of the total number of microglia counted per image to calculate percent phagocytosis.

##### 3.4.5.2 Quantifying phagocytic index

1. Import images into ImageJ (Fiji) as described above.
2. Select the Cell Counter plugin, and count the number of ingested neurons, which includes all neurons in contact with and inside phagocytic microglia.
3. Determine the phagocytic index as a measure of the average number of neurons phagocytosed per microglia, calculated with this formula

#### 3.4.6 Assessment of phagocytosis by live timelapse imaging

**Day 1**

1. Seed feeder cells into a 6-well tissue culture plate, add complete neuroblastoma culture media, and incubate at 37 °C, 5% CO_2_, and 70% humidity for 24h.

**Day 2**

1. UV-irradiate the cells for 15 minutes with a UV transilluminator (44 J/cm^2^), covering with aluminum foil.
2. Incubate the UV-irradiated cells at 37 °C, 5% CO_2_, and 70% humidity for 24h.

**Day 3**

1. Replace the culture media with 1 ml of PBS, and stain the cells with pHrodo at a concentration of 1 μg/ml.
2. Gently wash the stained cells with room-temperature PBS, then invert the plate to drain the PBS.
3. Add 1 ml of microglia culture media without M-CSF to the stained cells.
4. Harvest primary mouse brain microglia as described in section *3.2*.
5. Seed the isolated cells into each well containing UV-irradiated-labeled apoptotic neurons.
6. Incubate the cell mixture at 37 °C, 5% CO_2_, and 70% humidity for 24h. Live imaging was delayed for 4h, followed by an image every 5 minutes.

##### 3.4.6.1 Mean intensity value (for pHrodo) as a measure of phagocytosis

1. Upload image files into ImageJ (Fiji).
2. Using the negative control images, select Image>Adjust>Brightness/Contrast and use the “minimum” slide bar to define pHrodo baseline signal (should be barely visible). Record the corrected minimum signal value.
3. Select Analyze>Set measurement>select mean gray value and click apply.
4. Measure the mean intensity value for the image by selecting Analyze> Measure and record the mean intensity value.
5. Repeat for all images, adjusting the minimum slide bar values to the level used for the control images and measuring the mean intensity value as described in step 4.

## 4 Results

### 4.1 Exposure of phosphatidylserine residues by UV-irradiation induced apoptosis in the neuroblastoma cell line

We initially optimized parameters to efficiently induce apoptosis in our feeder cell type, the neuroblastoma cell line. We found that 15 min of UV exposure (44 J/cm^2^) followed by a 24h incubation resulted in extensive induction of apoptosis.

To assess the degree of apoptosis and exposure of PS, two experimental groups were used: (1) an untreated control group consisting of neuroblastoma cells cultured without UV exposure, and (2) a UV-treated group where neuroblastoma cells were exposed to UV light for 15 min and cultured for 24h post-irradiation. Cells from both groups were stained with Annexin V-FITC and propidium iodide to assess the extent of cell death and PS exposure. Unstained, single-stained Annexin V-FITC, and single-stained propidium iodide (Texas Red) controls were used to set gates (Supplementary Figure 1).

**Figure 2:**
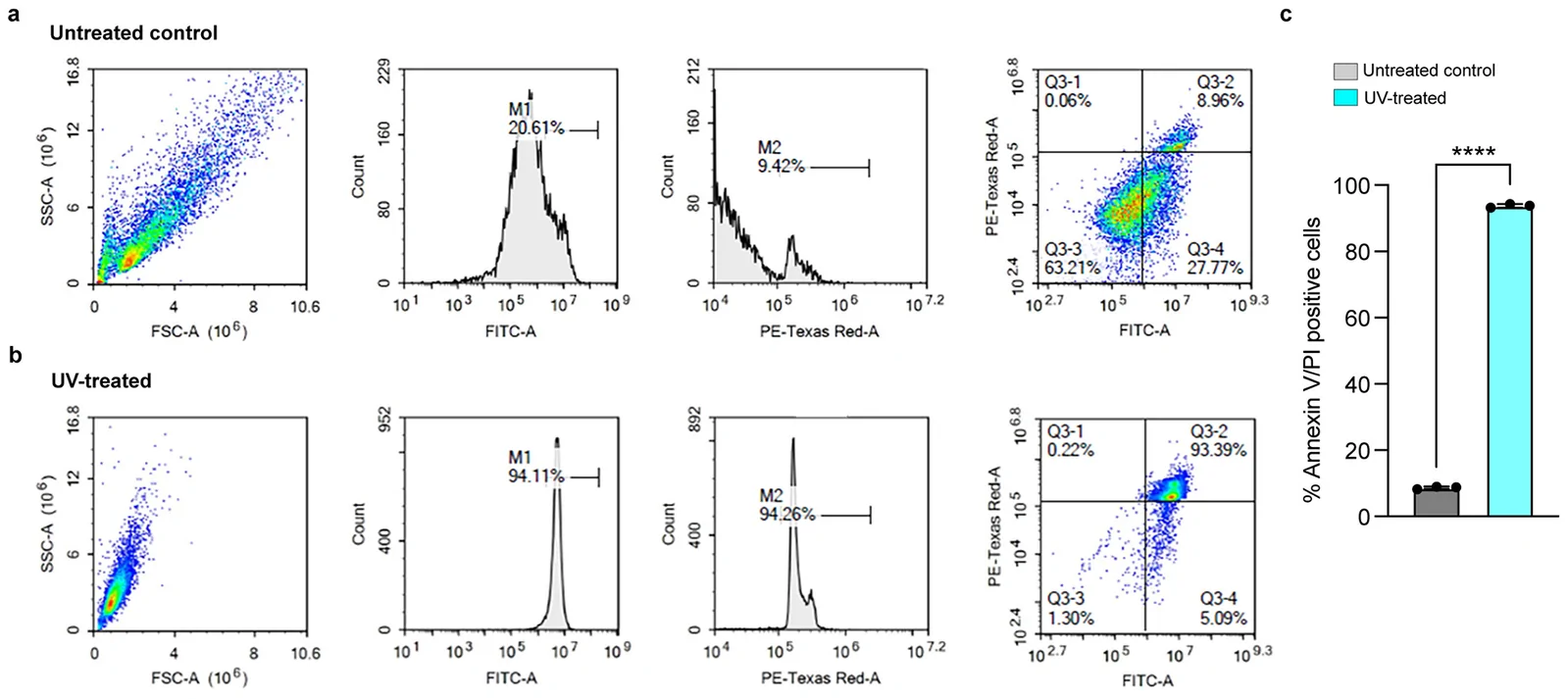
UV-irradiated cells undergo apoptosis and expose PS residues. **(a)** Non-irradiated control cells. **(b)** Cells irradiated with UV light. **(c)** Bar graph of Annexin V-FITC positive cells expressed as a percentage of the total number of cells analyzed. Data are presented as the mean ± SD. ****P < 0.0001 by Student's *t*-test (*n* = 3 biological replicates per condition).

Flow cytometry analysis demonstrated that UV irradiation of neuroblastoma cells for 15 min followed by a 24h incubation resulted in up to 96% cell death and PS exposure as indicated by Annexin V-FITC binding (Figure 2).

Following induction of apoptosis in the feeder cells using the established settings, the apoptotic cells were added to the isolated mouse brain microglia as detailed in procedure *3.4.4* and analyzed by FACS 24h later.

### 4.2 Assessment of phagocytosis by fluorescent activated cell sorting (FACS)

To assess *in vitro* microglial phagocytosis by FACS, primary mouse brain microglia (CX3CR1-EYFP) were isolated and seeded with UV-irradiated neuroblastoma cells labeled with the dead cell dye NHS-405. Microglia were mixed with the apoptotic labeled neurons at a 2:1 ratio in a cell culture vessel and incubated for 24h with gentle shaking (Figure 1).

The experimental groups were as follows: (1) Untreated, where dead neurons were not coated with Annexin V; and (2) treated, where dead neurons were pre-coated with Annexin V prior to mixing with the EYFP-expressing microglia. Single-stained controls for EYFP and Alexa Fluor^TM^ 405 were used to set individual gates before analyzing the test samples. Phagocytic cells were defined as double-positive for both Alexa Fluor^TM^ 405 and EYFP, whereas non-phagocytic cells were positive for EYFP only. Following sorting, these two cell populations were collected into separate tubes for downstream analyses.

**Figure 3:**
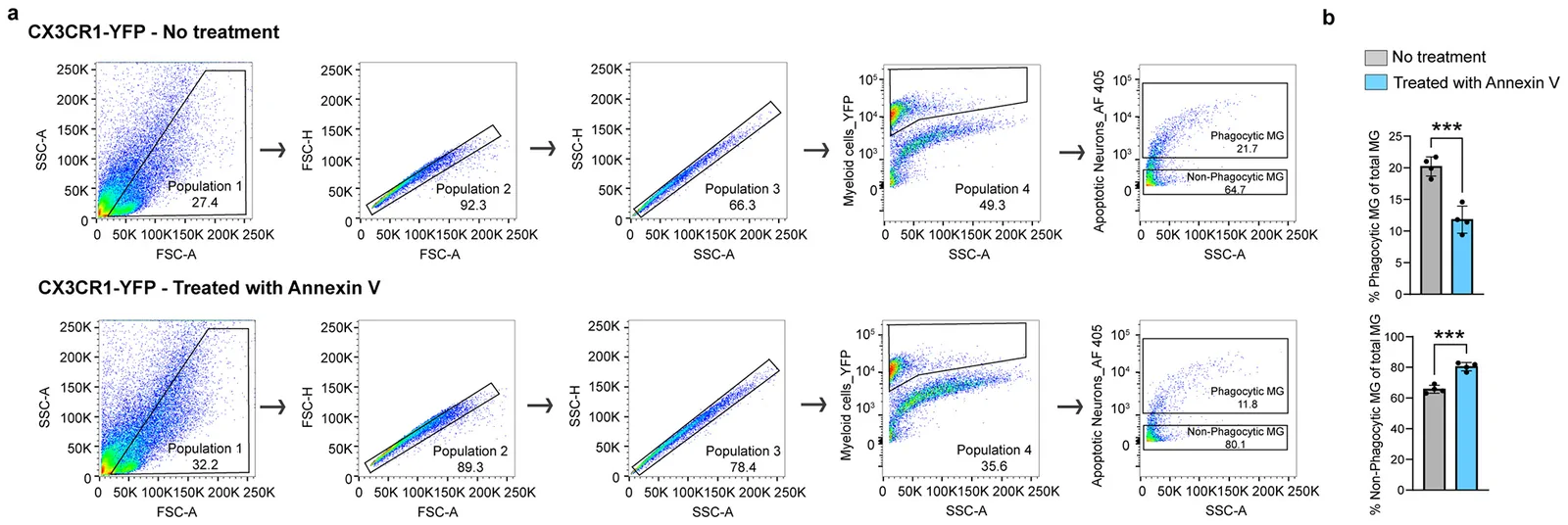
**(a)**. FACS plots showing phagocytic microglia and non-phagocytic microglia populations from 2-month-old CX3CR1-EYFP mice treated with apoptotic neurons with or without Annexin V. **(b)**. Quantification of the percentage of phagocytic microglia within the total microglia population treated with neurons with or without Annexin V. ***P < 0.0001 by Student's *t*-test (*n* = 4 biological replicates per condition). Data are presented as mean ± SD.

Analysis of the FACS data using FlowJo revealed a significant reduction in the proportion of phagocytic microglia in the Annexin V pre-treated group compared to the untreated group (Figures 3a, b). These findings indicate that UV-induced apoptosis, accompanied by PS exposure, facilitates microglial phagocytosis of apoptotic neurons. Notably, the most robust FACS results were obtained when intrinsically labeled microglia (CX3CR1-EYFP) were used, rather than microglia labeled with fluorescent antibodies.

As Cx3cr1 is a myeloid-specific marker, and the vast majority of myeloid cells in the brain are microglia in healthy adult mice (Silvin et al., 2022; Chhatbar et al., 2026), we expect most of our YFP^+^ cells to be microglia. Flow cytometry analysis of the YFP^+^ cell population revealed that 12% of YFP^+^ cells were positive for the microglia-specific marker Tmem119 upon isolation (Supplementary Figure 2). While lower than expected, this may be the result of incomplete staining with this antibody or the rapid degradation of the Tmem119 protein due to activation caused by isolation conditions. Flow cytometry showed that Tmem119 expression is completely abolished following 24-h co-culture with dead neurons, which suggests further activation.

To address potential concerns regarding the death of microglia during the phagocytosis assay, we stained for the cell death marker propidium iodide before and after 24-h co-culture with dead neurons (Supplementary Figure 3). We found approximately 10% propidium iodide positivity in the YFP^+^ population immediately upon isolation and following 24-h co-culture with dead neurons. This suggests that only a modest amount of microglial cell death occurs during the phagocytosis assay.

The *in vitro* FACS-based assessment of phagocytosis described here is a rapid and effective method which enables downstream analysis of sorted cell populations using techniques such as bulk RNA sequencing, qPCR, and Western blotting. These downstream analyses could be used to evaluate the role of molecules involved in the phagocytic pathway.

### 4.3 Assessment of phagocytosis by ICC

Next, microglial phagocytosis of apoptotic neurons was verified by ICC. For this assay, primary mouse brain microglia were isolated and cultured in EZ slide chambers for up to 5 days. This allowed sufficient time for the microglia to adhere firmly to the slide, ensuring they could withstand the rigorous cell staining protocol.

UV-irradiated neurons were labeled with the dead cell dye Alexa Fluor^TM^ 405 prior to being added to the cultured microglia. Microglia were seeded either with dead neurons alone, or dead neurons pre-coated with Annexin V. The cell mixture was incubated for 24h.

**Figure 4:**
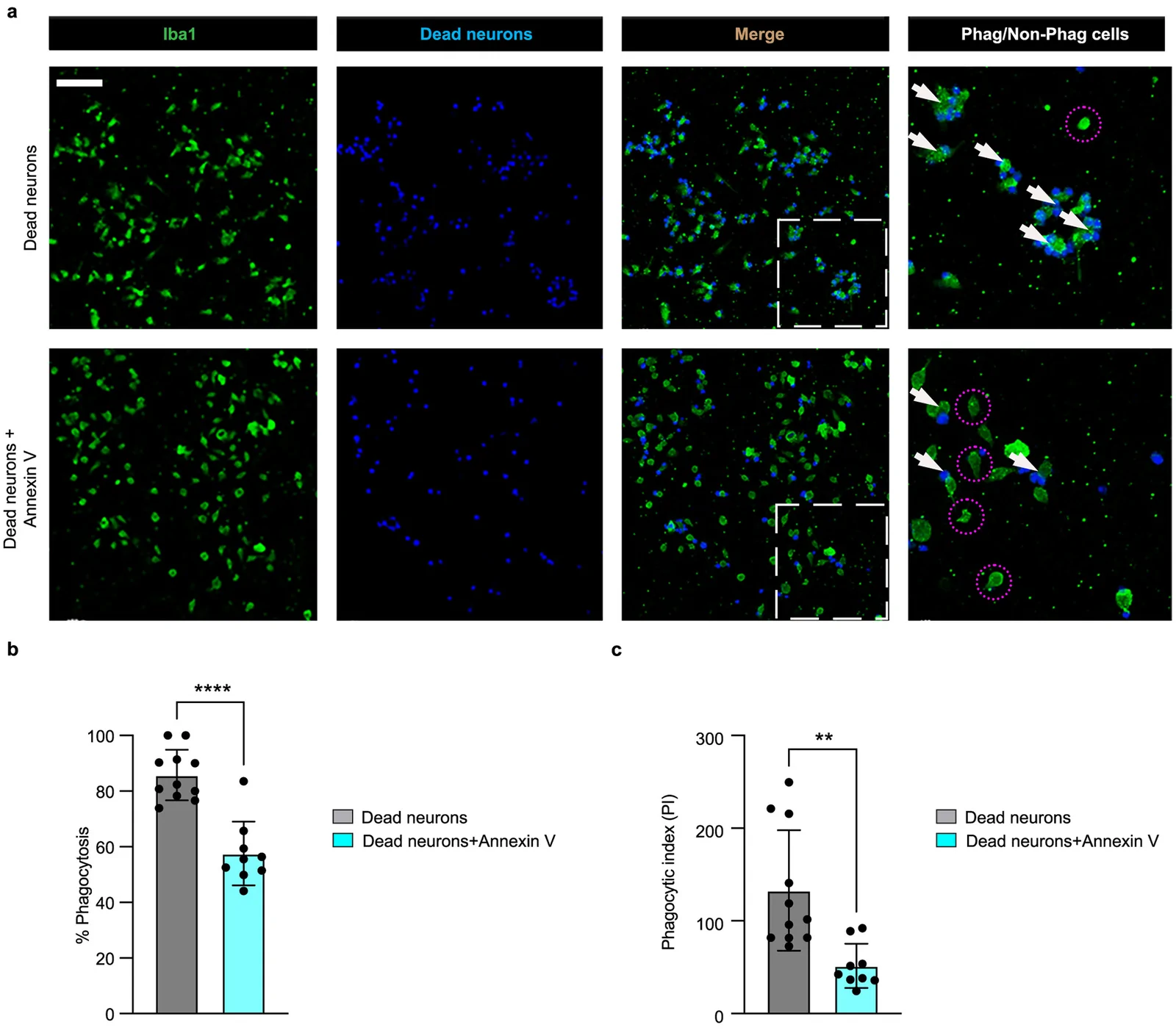
PS-mediated phagocytosis of apoptotic neurons *in vitro* is blocked by Annexin V. **(a)**. Micrographs showing microglia in green and apoptotic neurons in blue. White arrowheads indicate phagocytic microglia, while purple circles highlight non-phagocytic microglia. Scale bar 200 μm. Imaged on 10 x objective. **(b)**. Percentage of dead neurons phagocytosed by microglia with and without Annexin V. **(c)**. Microglial phagocytic index of dead neurons with and without Annexin V. Data in panels b and c represent mean ± SD from three biological replicates with 3–5 technical replicates each. ****P < 0.0001, **P ≤ 0.01 by Student's *t*-test.

Following incubation, standard washing, blocking, and permeabilization steps were performed. The cells were then stained with the pan-myeloid marker Iba1 and visualized using an Alexa Fluor 488-conjugated secondary antibody (Figure 4a). Representative images were captured at 20 × magnification, and phagocytosis was quantified from the images. Ultimately, there was a reduction in phagocytosis measured both by percent phagocytosis and phagocytic index when apoptotic neurons were pre-coated with Annexin V prior to their addition to the microglial culture (Figures 4b, c).

To explore the reproducibility of phagocytosis quantification across different dead cell dyes, ICC phagocytosis measurements were replicated using the pH-sensitive apoptotic cell dye, pHrodo. Notably, the permeabilization step of ICC abolishes any localized pH differences, which leads to uniform pHrodo fluorescence in ICC. A reduction in both phagocytic capacity and phagocytic index was observed when pHrodo-labeled apoptotic neurons were pre-coated with Annexin V before their addition to cultured microglia (Supplementary Figures 4a–c). Importantly, there were no differences observed between data generated using NHS-405 and pHrodo (Supplementary Figures 4d, e), indicating consistency across labeling approaches. As described here, ICC-based assessment of phagocytosis represents a robust and complementary analytical method, providing visual evidence of phagocytic events. Moreover, it yields quantitative metrics such as the percentage of phagocytic microglia and the phagocytic index, which can be highly informative for elucidating the roles of specific microglial genes in the regulation of phagocytosis.

Finally, we also explored the expression of microglial homeostatic and activation markers in cell culture. After incubating the cell isolates for 5 days in culture with M-CSF, nearly 100% were positive for Tmem119 via ICC staining, while P2ry12 expression was detectable but very low (Supplementary Figure 5). We also checked expression of DAM markers Apoe, Galectin-3, and Osteopontin (OPN), which are all upregulated in brain and retinal microglia following phagocytosis of dead neurons *in vivo* (Krasemann et al., 2017; Margeta et al., 2022) (Supplementary Figure 5). All DAM markers were upregulated in microglia both before and after co-culture with dead neurons (Supplementary Figure 6). DAM marker expression was not different between phagocytic and non-phagocytic microglia, further suggesting that microglia are basally activated in culture.

### 4.4 *In vitro* timelapse imaging of microglia phagocytosis of dead neurons labeled with the pH sensitive dye pHrodo

To further validate ICC, *in vitro* live time-lapse imaging was performed using the pH-sensitive dye pHrodo. The fluorescence of pHrodo increases in response to low pH, such as when a labeled cell is internalized by microglia in the phagolysosome (Supplementary Figure 7; Supplementary Video S1). For the live imaging assay, feeder cells were grown in a culture vessel, irradiated with UV light, and labeled with pHrodo. Freshly isolated primary mouse brain microglia were seeded on top of the dead, labeled neurons pre-treated with or without Annexin V, and the cell mixture was incubated at 37°C and 5% CO_2_. Timelapse images were taken on an EVOS 7,000 microscope with on stage incubator (Thermo Fisher Scientific, MA, USA) at 37°C, 5% CO_2_, and 70% humidity. The well containing neuroblastoma cells only was used to measure baseline pHrodo fluorescence.

**Figure 5:**
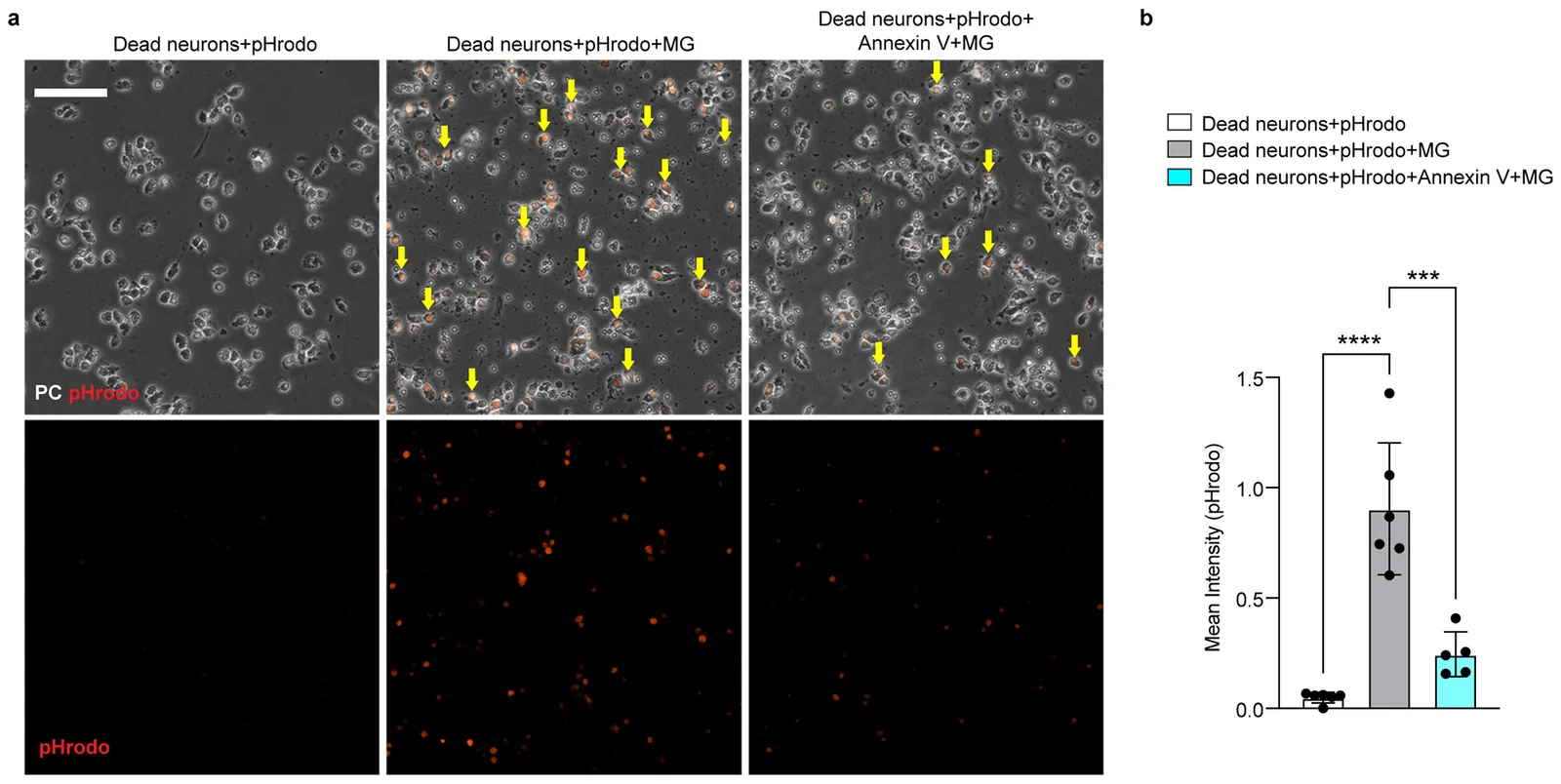
Representative phase contrast and pHrodo image sequences at the 4h timepoint of *in vitro* time-lapse imaging showing microglial phagocytosis of pHrodo-labeled apoptotic neurons. **(a)** Micrographs showing phase contrast and pHrodo images for each treatment. Scale bar 50 μm. Imaged on 20x objective. **(b)**. Bar graph of pHrodo mean intensity values. Data represent mean ± SD from three biological replicates with two technical replicates each. ****P < 0.0001, ***P ≤ 0.001 (One-way ANOVA).

Time-lapse imaging was initiated after a 4-h delay, allowing the microglia to settle to the bottom of the culture vessel near the dead neurons. Following this incubation period, images were captured every 5 min, with six replicates per treatment group. Notably, pHrodo fluorescence only decreased after the initial 4h delay period, which suggests that phagocytosis predominantly occurred in the first 4 h. Therefore, still images captured at the 4-h timepoint were used to assess phagocytosis by measuring the mean pHrodo fluorescence intensity. Analysis of the pHrodo mean intensity values revealed that Annexin V pre-treatment of dead neurons significantly inhibited microglial phagocytosis, as indicated by a reduction in fluorescence intensity in the Annexin V-treated group (Figures 5a, b).

**Figure 6:**
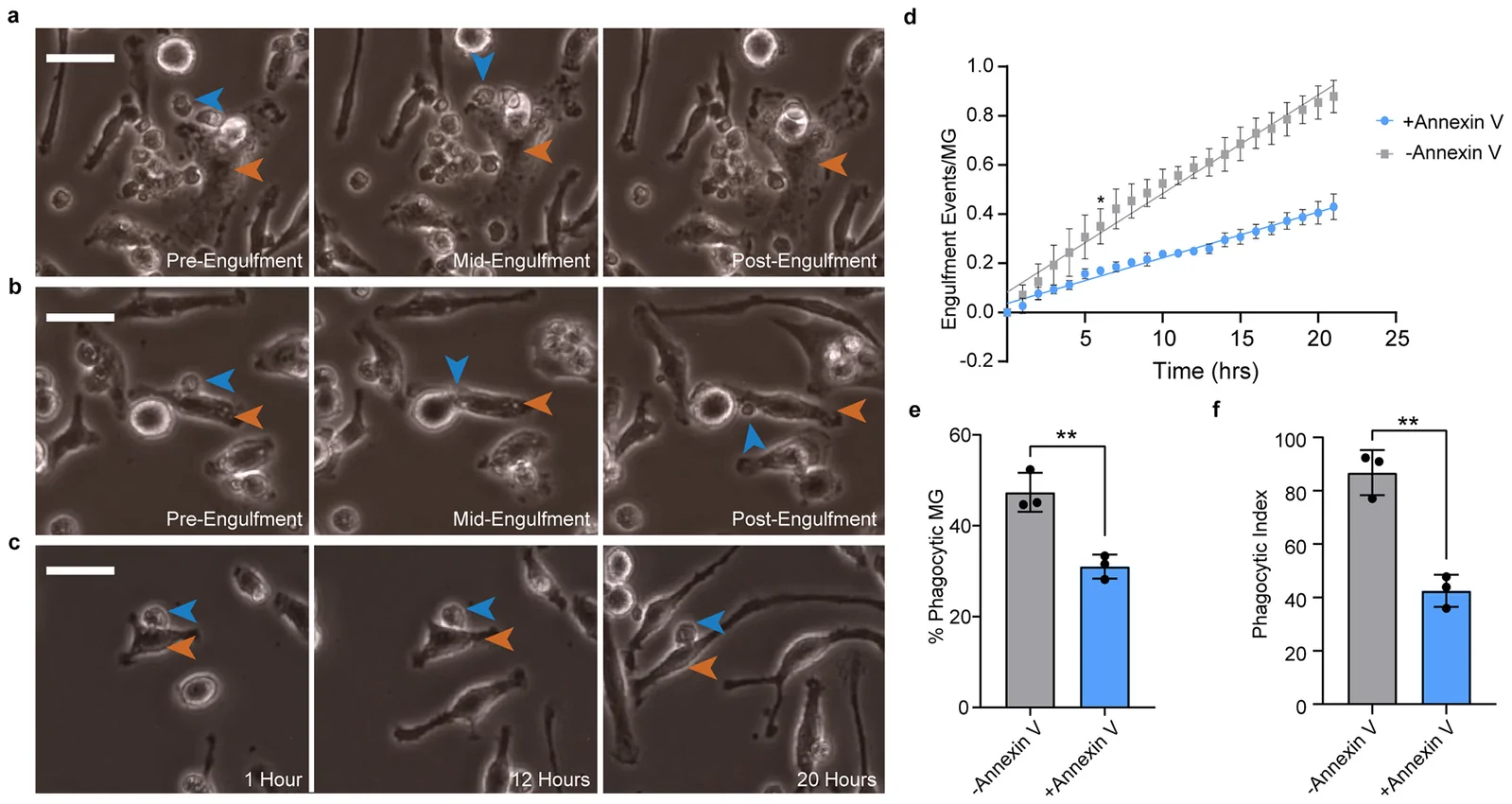
Annexin V treatment decreases microglial phagocytosis assessed via live imaging. **(a–c)** Representative phase contrast images of microglial phagocytosis of UV-irradiated neuroblastoma cells. Dead neurons of interest are marked with a blue arrow, and microglia of interest are marked with an orange arrow. **(a)** A complete engulfment event occurs when microglia engulf a dead neuron, and the dead neuron does not re-emerge. Scale bar 25 um. Imaged on 20x objective. **(b)** A partial engulfment event occurs when microglia appear to engulf a dead neuron, and a smaller part of the dead neuron re-emerges. Scale bar 15 um. Imaged on 20x objective. **(c)** A dead neuron that is attached to microglia but does not decrease in size has not been engulfed. Scale bar 15 um. Imaged on 20x objective. **(d)** Plot of total engulfment events per microglia over time. Total engulfment events per microglia are different between treatment with and without Annexin V after the 6-h timepoint, as denoted by the “*” symbol. **(e)** Bar graph of the percentage of phagocytic microglia for each treatment. **(f)** Bar graph of phagocytic index for each treatment. Data represent mean ± SD from three technical replicates. **P ≤ 0.01, *P ≤ 0.05 (Student's *t*-test).

To demonstrate that phagocytosis can be monitored without fluorescent dyes, we replicated the above time-lapse imaging experiment using phase-contrast microscopy. This approach allows the study of microglial phagocytosis in laboratories equipped with time-lapse imaging systems lacking fluorescence live-imaging capabilities. UV-irradiated neuroblastoma cells were added to cultured primary mouse brain microglia, and phase-contrast images were captured every 2 min over a 21-h period. Without fluorescent labeling, individual engulfment events were visually identified and manually quantified from the recorded videos (Figures 6a–c; Supplementary Videos S2–S3). We found that microglia engulf Annexin V–treated neuroblastoma cells at a lower rate than untreated cells (Figure 6d). Consistent with the FACS and ICC results, Annexin V treatment reduced both percent phagocytosis and phagocytic index of microglia (Figures 6e, f).

## 5 Discussion

### 5.1 Conclusions

We describe here a straightforward and robust multi-assay protocol for the *in vitro* evaluation of phagocytosis. This workflow can be performed using either freshly isolated primary cells or established cell lines, making it adaptable to a wide range of experimental systems. Importantly, it relies primarily on standard laboratory equipment readily available in most research facilities, ensuring broad accessibility without specialized instrumentation. The combination of FACS, ICC, and *in vitro* live-cell imaging provides complementary and mutually reinforcing perspectives on phagocytic activity. FACS allows rapid, quantitative assessment of phagocytic efficiency across large cell populations; ICC enables detailed visualization of microglial morphology and cell–cell interactions; and time-lapse imaging captures the kinetics and dynamics of engulfment in real time. Since these methodologies are complementary, this protocol offers multiple approaches to study microglial phagocytosis of neurons which can be flexibly implemented depending on available resources. In doing so, it enables laboratories to leverage their existing technologies without the need to establish entirely new workflows.

Beyond functional assessment, this integrated approach can also be used for downstream molecular analyses, including bulk RNA sequencing, qPCR, and Western blotting of sorted microglial populations. Such analyses can provide valuable insights into the transcriptomic and proteomic changes associated with active engulfment and reveal key molecular regulators of phagocytic pathways. However, it is important to note that the process of cell isolation and cell culture activates microglia, and that therefore, their transcriptional responses may not be identical to what is observed *in vivo*. As evidence of this, we show that microglia in culture express key DAM markers in the absence of dead neurons (Supplementary Figures 5, 6). This apparent basal activation of primary microglia in culture should be considered when assessing their transcriptional responses following phagocytosis. It is also important to note that the YFP^+^ cells that we isolate from Cx3cr1-YFP animals likely contain a small fraction of border-associated macrophages (BAMs) in addition to microglia. Users of this protocol wishing to ensure 100% microglial purity for their assays can take advantage of *Tmem119*^*CreERT*2^ *Ai14* or *P2ry12*^*CreERT*2^ *Ai14* reporter lines, in which only microglia will be labeled with tdTomato following tamoxifen administration to donor animals (Kaiser and Feng, 2019; McKinsey et al., 2020). Finally, our protocol used whole brain isolation to establish a generalizable method for *in vitro* quantification of microglial phagocytosis. However, when reproducing this assay using region-specific isolation, it is important to consider that microglia have diverse activation phenotypes, influenced by disease-associated signaling, stimuli, species, and local microenvironment (Pérez-Boyero et al., 2025; Farias Quipildor et al., 2026; Michels et al., 2026). Thus, microglial heterogeneity across brain regions can impact baseline activation state and their phagocytic capacity. Of note, the same experimental approach can also be used to isolate and culture retinal microglia, although this would require significant pooling of retinas to obtain appropriate cell counts (Margeta et al., 2022). Though our current protocol cannot differentiate between microglial subpopulations in the retina, this limitation may be addressed through complementary approaches such as spatial transcriptomics and retinal explant assays.

Importantly, our protocol provides a physiologically relevant alternative to existing *in vitro* phagocytosis assays. While current assays, including Zymon-based, bead-based, pHrodo-based, and nanoparticle-based assays, offer insight into microglial phagocytic mechanisms, they rely on artificial, bacterial, or yeast particles that do not fully reflect the neurodegenerative microenvironment. The utilization of labeled apoptotic neuroblastoma cells in our protocol as a phagocytic target offers a more biologically accurate model. However, the use of human SH-SY5Y neuroblastoma cells with primary mouse microglia creates a cross-species assay which could increase reactivity, serving as a potential limitation. Users wishing to minimize this issue can consider culturing primary mouse neurons and inducing apoptosis using UV light exposure as described previously (Krasemann et al., 2017).

Altogether, this accessible and versatile framework provides a powerful means to investigate the cellular and molecular mechanisms of microglial phagocytosis—a process central to neural development, immune regulation, and the pathogenesis of numerous neurodegenerative diseases.

### 5.2 Additional considerations and potential adjustments

1. After UV irradiation of the feeder cells, gentle tapping of the flask may be required to resuspend the cells prior to the 24h incubation.
2. While we used a ratio of microglia to neurons of 2:1, as suggested by Cosker et al. (2021), alternative ratios may be used as relevant to the specific experimental question. The work by Chu et al. (2020) exploring the effects of seeding ratios on macrophage phagocytosis gives some idea of what an optimal seeding ratio may be for microglia.
3. An alternative enzyme such as Collagenase A (Roche, #11088793001) can be used to detach cells after phagocytosis to improve cell recovery.
4. We used the CX3CR1-EYFP line for the isolation of mouse brain microglia for FACS. Using endogenously labeled cells is not a requirement to execute this protocol. Wild-type microglia labeled with a combination of fluorescent antibodies can also be used, but we found this approach to be more variable.
5. It is recommended to pre-block all tubes used for cell preparation with blocking buffer (2% BSA) for 30 min.
6. For the FACS data, phagocytosis was allowed to proceed for 24h; however, this duration can be adjusted to suit the scientific question being investigated. In our case, we wanted to give the cells enough time to develop a detectable gene expression profile following phagocytosis.
7. For phagocytosis assessment by ICC, a five-day waiting period was used to allow the cells enough time to properly adhere to the slide. This was important to ensure that the cells could sustain the rigorous ICC staining procedure that involves multiple solution changes, which could otherwise cause cell loss due to detachment if adequate adherence time is not provided. However, a 24h waiting to mimic the FACS data could be tested. This approach would require several protocol adjustments, such as coating culture plates with a substrate to enhance cell attachment or increasing the initial seeding density of microglia.
8. When analyzing the mean intensity values of images in ImageJ, for images with a scale bar embedded one should exclude the scale bar while defining the area to be analyzed. The scale bar produces an artificial increase in the image's mean intensity.
9. In the ICC phagocytosis assay, we assume all microglia that are attached to dead neurons are phagocytic because we have only blocked the binding and recognition steps of phagocytosis using Annexin V. Thus, any attached dead neuron is assumed to be in the process of internalization. However, these ICC criteria can be adjusted by future protocol users based on their experimental questions.

## Data Availability

The original contributions presented in the study are included in the article/Supplementary material, further inquiries can be directed to the corresponding author.
