# Supplementary material for: Assessing microglial phagocytosis of apoptotic neurons in vitro: a multimodal approach
Source: Front Neurosci. 2026 Aug 12;20:1813556. doi: 10.3389/fnins.2026.1813556 (PMC13506744; doi:10.3389/fnins.2026.1813556)
Supplement: Supplementary file 1 [file Data_Sheet_1.pdf]

Supplementary Material

1 Supplementary Figures

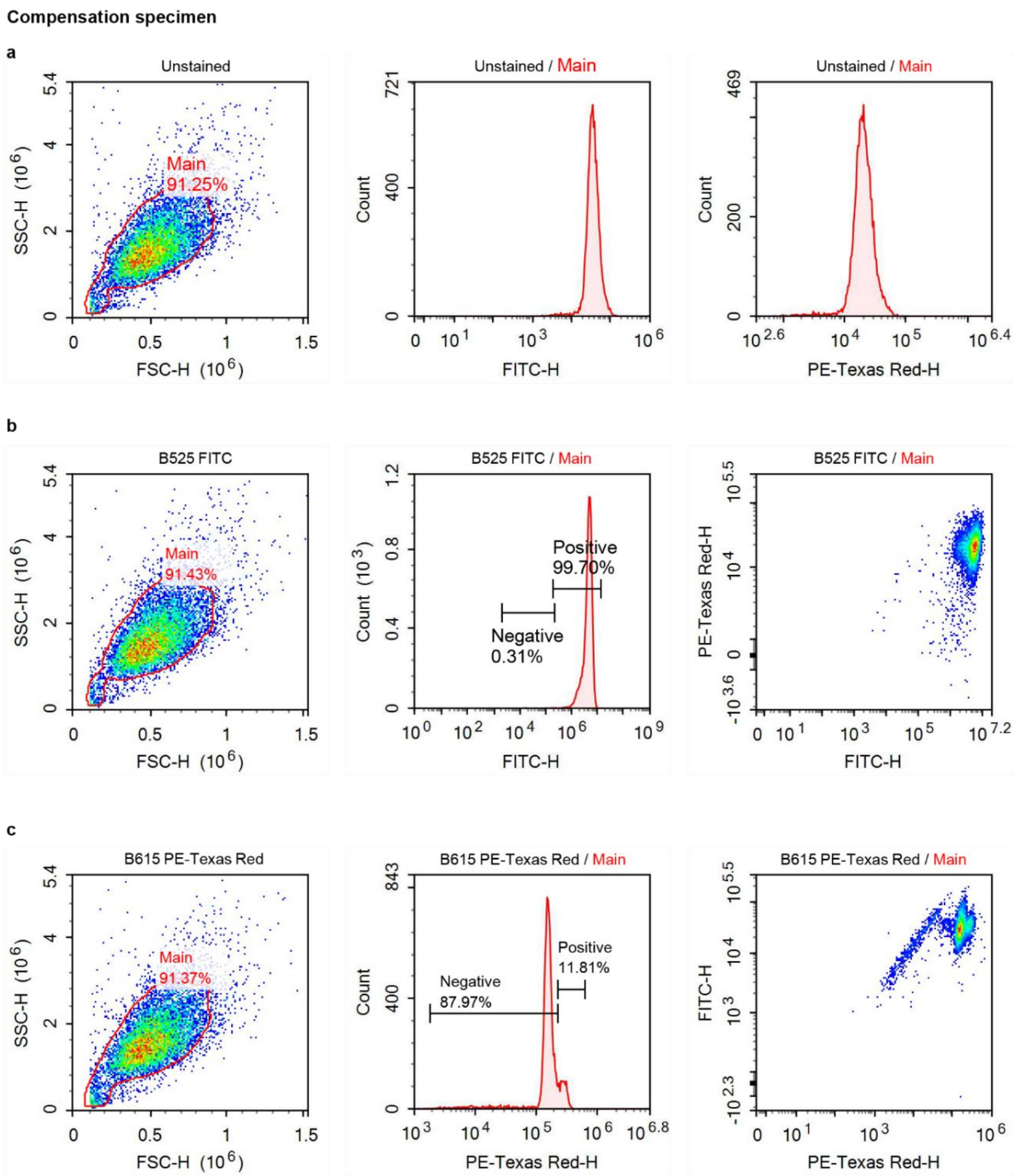

Supplementary Figure 1. Flow cytometry plots showing the compensations used prior to running the test samples. **(a)** is the unstained control, **(b)** is FITC-conjugated Annexin V and (c) is the PE-Texas Red for propidium iodide.

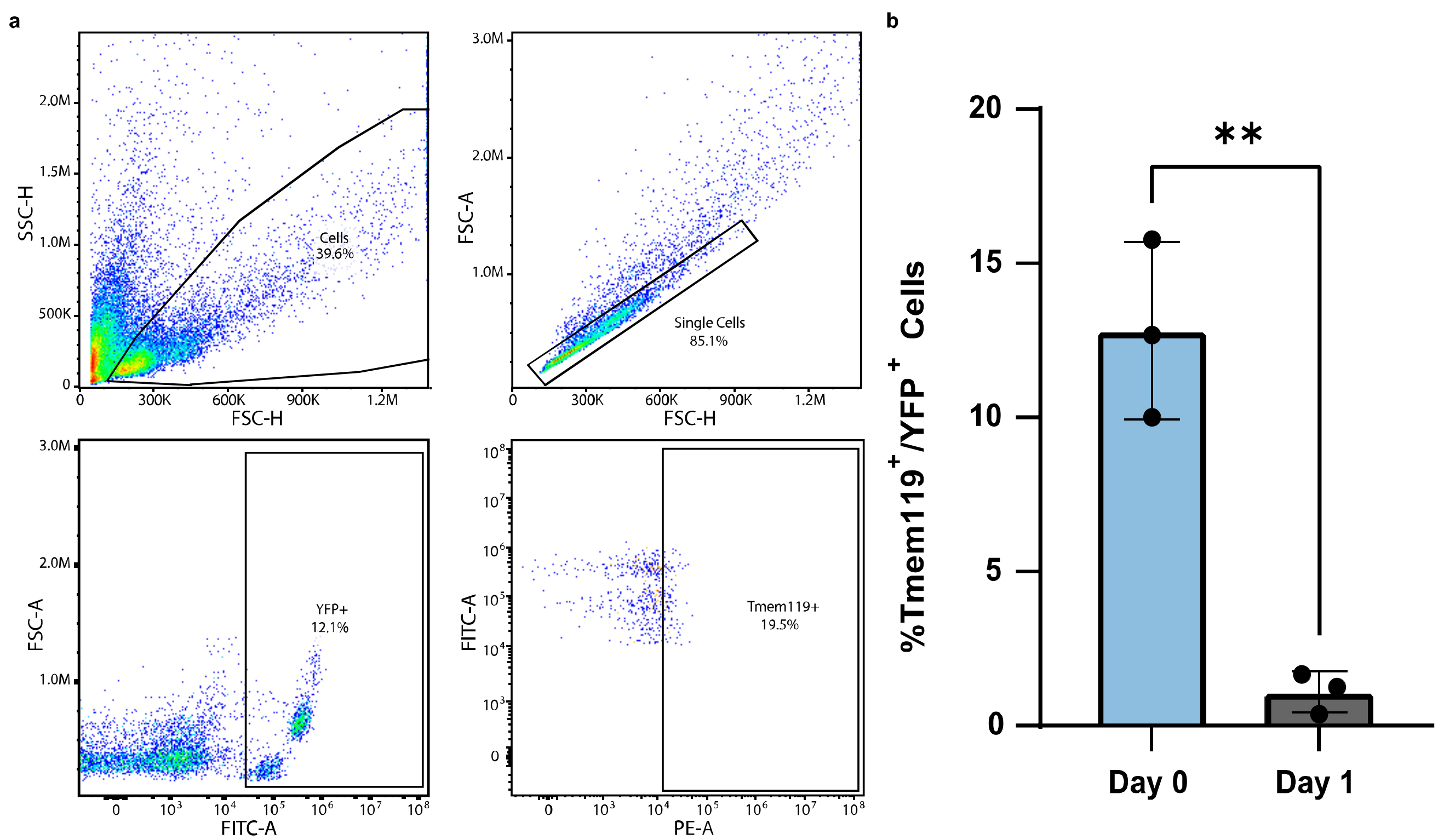

Supplemental Figure 2. A minority of Cx3cr1-YFP microglia are Tmem119 positive upon isolation, and they lose all Tmem119 positivity after 24-hour co-culture with dead neurons. Mouse brain isolates were sorted for YFP and Tmem119-PE. **(a)** Flow cytometry plots showing the gating strategy to determine Tmem119 positivity. **(b)** Bar graph depicting Tmem119 positivity of Cx3cr1-YFP microglia immediately upon isolation and following 24-hour co-culture with dead neurons. Data represent mean ± SD from 3 biological replicates with 3 technical replicates each. **P ≤ 0.01 by Student's t-test.

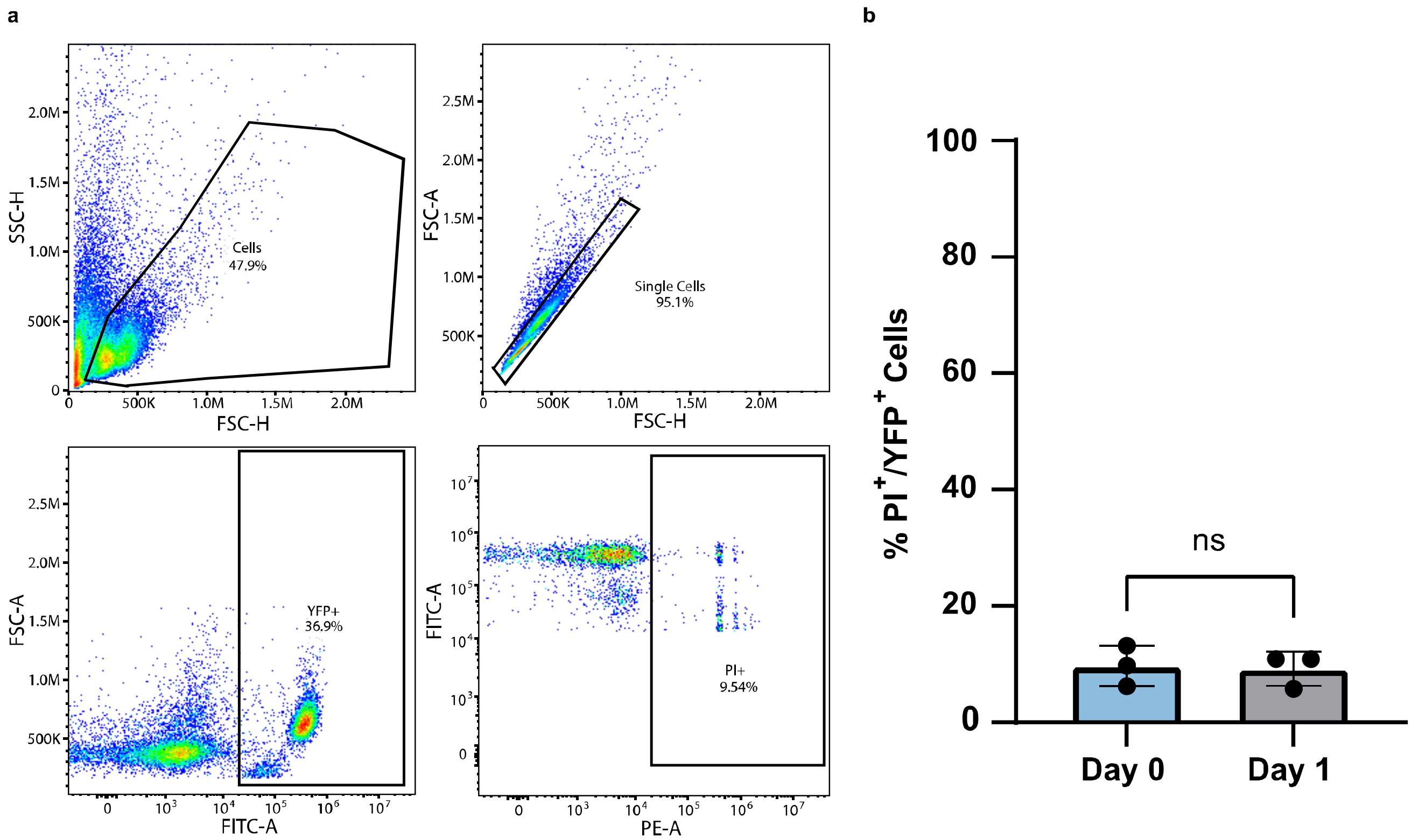

Supplemental Figure 3. Cx3cr1-YFP microglia show high viability upon isolation and after 24-hour co-culture with dead neurons. Mouse brain isolates were sorted for YFP and the cell death marker propidium iodide (PI). **(a)** Flow cytometry plots showing the gating strategy to determine PI positivity. **(b)** Bar graph depicting PI positivity of Cx3cr1-YFP microglia immediately upon isolation and following 24-hour co-culture with dead neurons. Data represent mean ± SD from 3 biological replicates with 3 technical replicates each. ns = not statistically significant by Student's t-test.

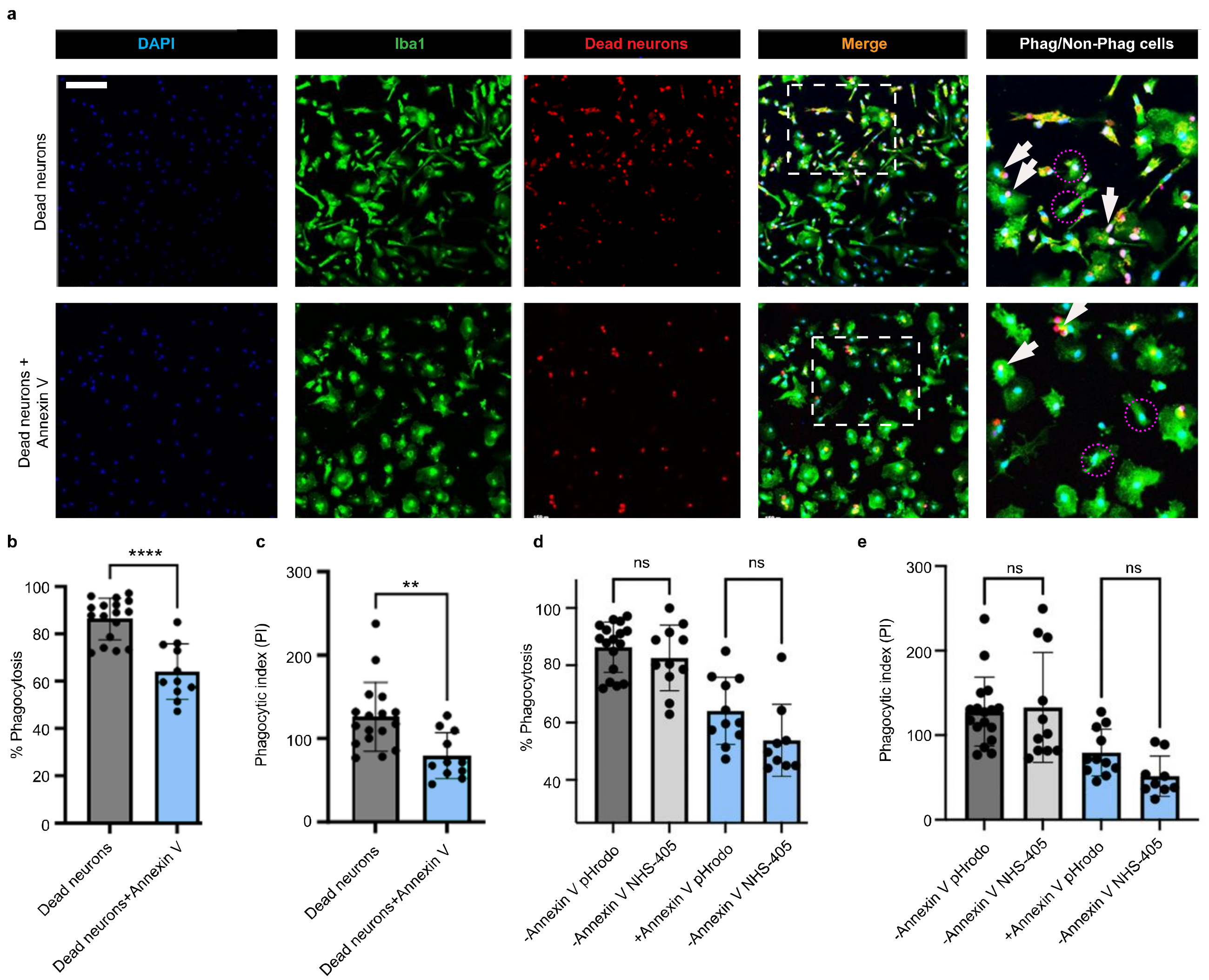

Supplementary Figure 4. PS-mediated phagocytosis of apoptotic neurons *in vitro* is comparable when using NHS-405 or pHrodo. (**a**). Micrographs showing microglia labeled in green and apoptotic neurons in red labeled with pHrodo (following permeabilization step during ICC). White arrowheads indicate phagocytic microglia, while purple circles highlight non-phagocytic microglia. Scale bar 200 µm. Imaged on 10x objective. (**b**)**.** Percentage of microglial phagocytosis of dead neurons labeled with pHrodo with and without Annexin V. (**c**). Microglial phagocytic index of dead neurons labeled with pHrodo with and without Annexin V. (**d**-**e**). There is no difference in percentage phagocytosis or phagocytic index when using cells stained with either dead cell dye 405 or pHrodo. Data represent mean ± SD from 3-4 biological replicates with 3-5 technical replicates each. ***P < 0.0001, **P ≤ 0.01 by Student's t-test. ns = not statistically significant.

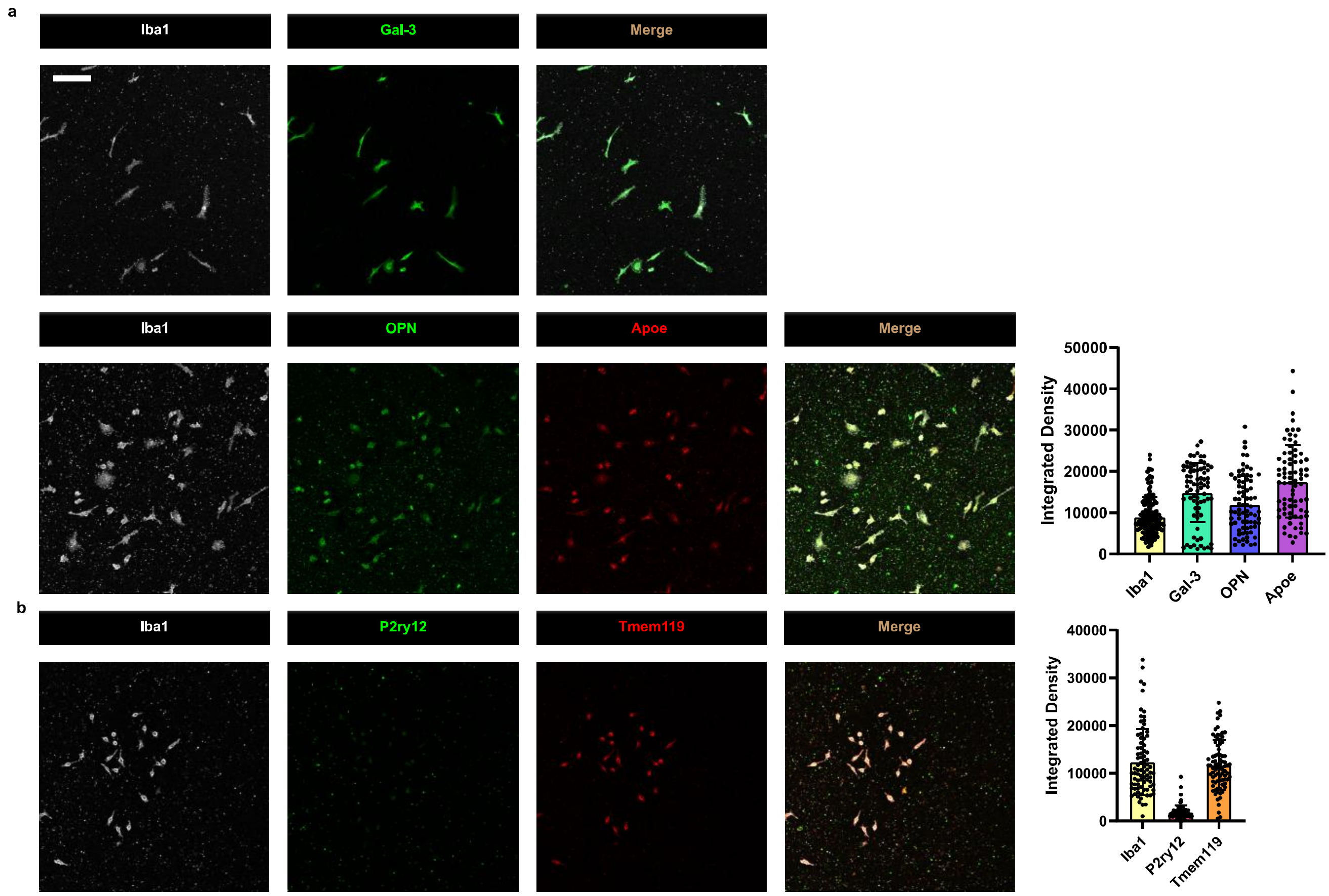

Supplemental Figure 5. Primary microglia in culture are basally activated. Micrographs depict naive microglia in culture stained for **(a)** disease-associated microglia (DAM) markers Galectin-3, OPN, and Apoe, and **(b)** homeostatic markers P2ry12 and Tmem119. Scale bar 200 µm. Imaged on 10x objective. Bar graphs represent the integrated density of fluorescence for each marker. Individual dots represent individual microglia. Data represent mean ± SD from 3 biological replicates with 3 technical replicates each.

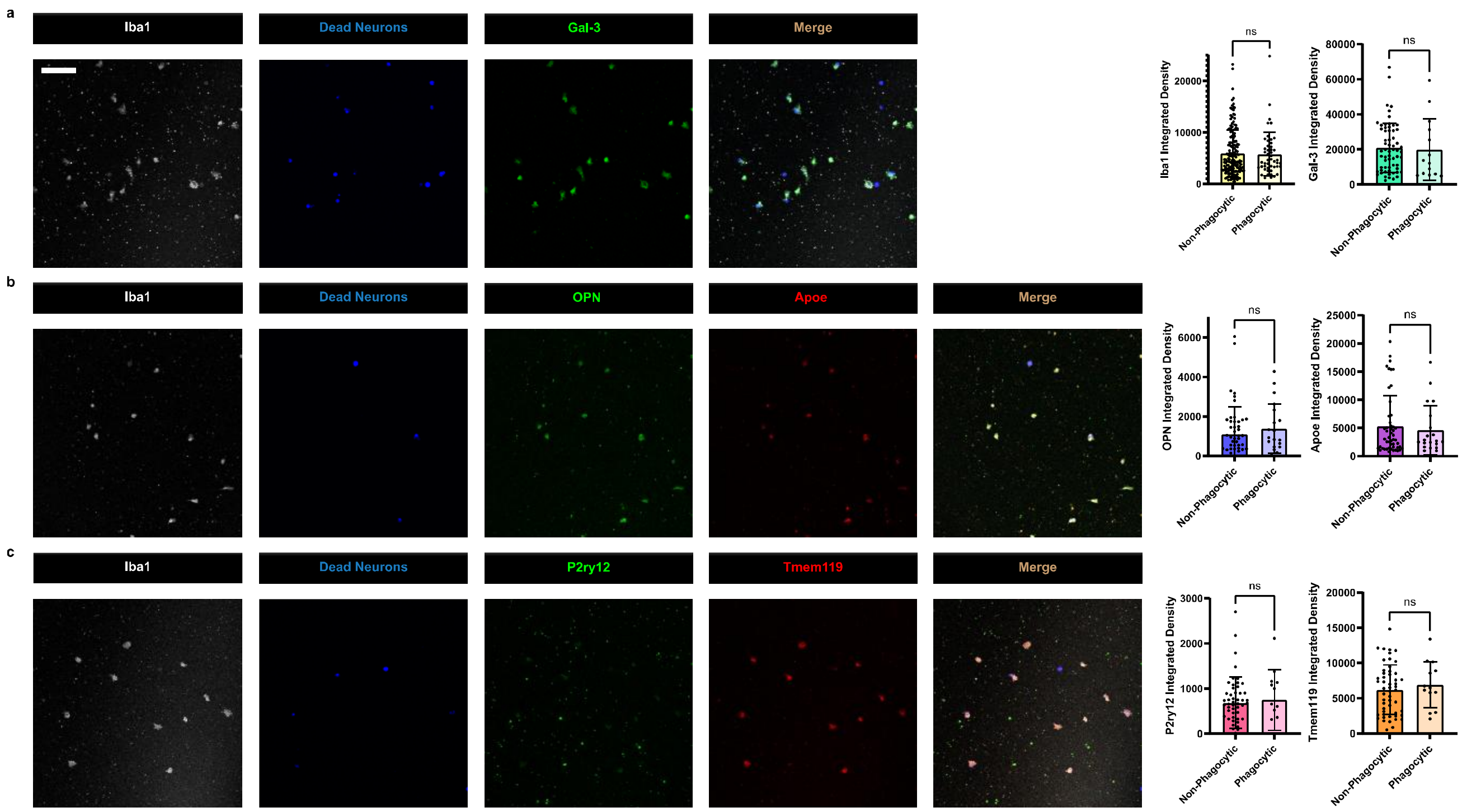

Supplemental Figure 6. Primary cultured microglia do not further upregulate disease-associated microglial genes following phagocytosis of dead neurons. Micrographs depict microglia in culture stained for **(a)** DAM markers and **(b)** homeostatic markers following 24-hour co-culture with dead neurons. Scale bar 200 µm. Imaged on 10x objective. Bar graphs represent the integrated density of fluorescence for each marker in phagocytic and non-phagocytic microglia after 24-hour co-culture with dead neurons. Phagocytic microglia were in contact with dead neurons, and non-phagocytic microglia were not in contact with dead neurons. Individual dots represent individual microglia. Data represent mean ± SD from 3 biological replicates with 3 technical replicates each. ns = not statistically significant by Student's t-test.

a

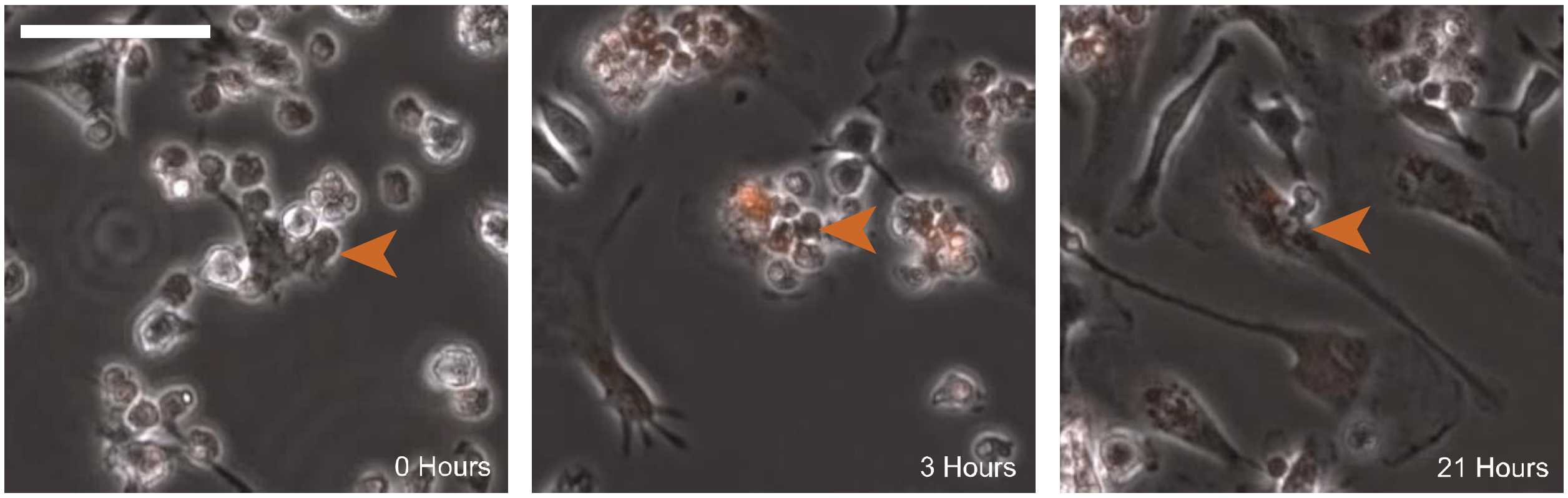

b

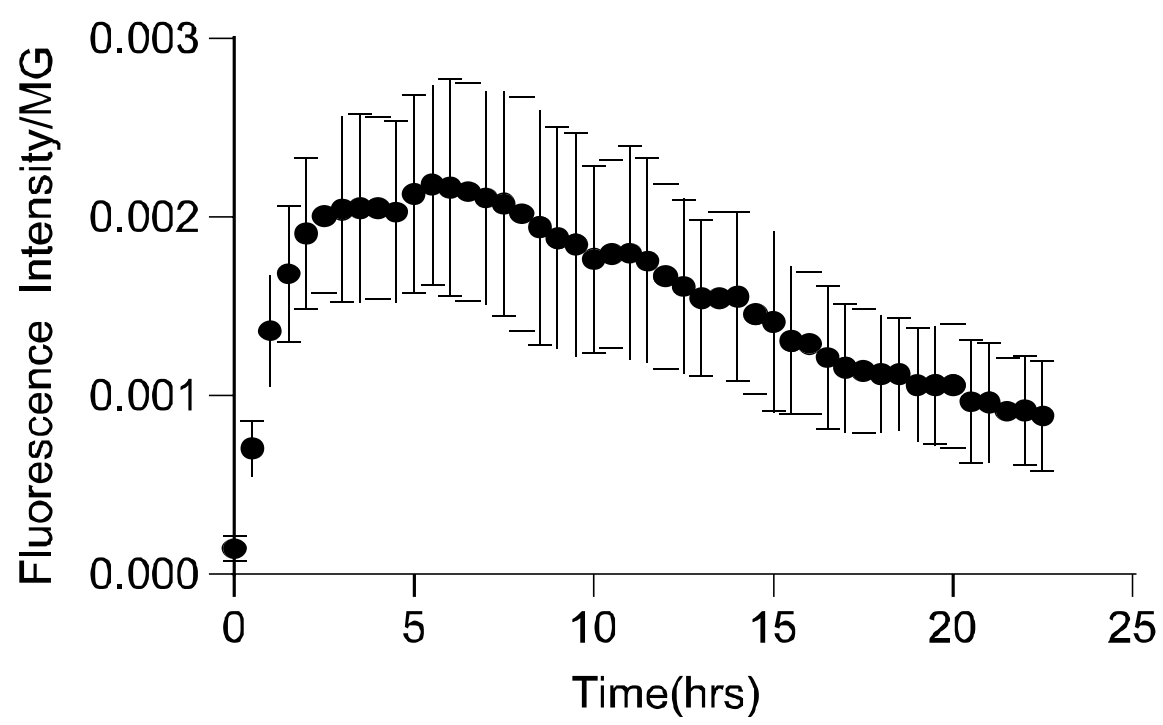

Supplementary Figure 7. Microglia fluorescence increases after engulfment of pHrodo-labeled apoptotic neuroblastoma cells. There is an initial increase in fluorescence intensity during phagocytosis onset, followed by a decline in fluorescence intensity as microglia clear phagolysosomes. (**a**) Representative images of a B6 microglia after seeding with pHrodo-labeled neuroblastoma cells. Orange arrows point to the microglia of interest. Scale bar 50 µm. Imaged on 20x objective. (**b**) Graph of average microglia fluorescence over time after seeding with pHrodo-labeled neuroblastoma cells. Fluorescence intensity per microglia was calculated by dividing the average fluorescence intensity by the number of microglia in each frame. Data represent mean ± SD from five technical replicates.

2 Supplementary Videos

Supplementary Video S1. Timelapse of microglial phagocytosis of pHrodo-labeled dead neurons. Microglia fluoresce upon engulfment of pHrodo-labeled dead neurons.

Supplementary Video S2. Representative phase-contrast timelapse of microglial phagocytosis of apoptotic neuroblastoma cells.

Supplementary Video S3. Representative phase-contrast timelapse of microglial phagocytosis of apoptotic neuroblastoma cells pre-treated with Annexin V.
